# Natural Products with Inhibitory Activity against Human Immunodeficiency Virus Type 1

**DOI:** 10.1155/2021/5552088

**Published:** 2021-05-29

**Authors:** Maria S. Serna-Arbeláez, Laura Florez-Sampedro, Lina P. Orozco, Katherin Ramírez, Elkin Galeano, Wildeman Zapata

**Affiliations:** ^1^Grupo Infettare, Facultad de Medicina, Universidad Cooperativa de Colombia, Medellín, Colombia; ^2^Grupo de Investigacion en Ciencias Animales-GRICA, Facultad de Medicina Veterinaria y Zootecnia, Universidad Cooperativa de Colombia, Bucaramanga, Colombia; ^3^Grupo Inmunovirología, Facultad de Medicina, Universidad de Antioquia (UdeA), Medellín, Colombia; ^4^Productos Naturales Marinos, Departamento de Farmacia, Facultad de Ciencias Farmacéuticas y Alimentarias, Universidad de Antioquia (UdeA), Medellín, Colombia

## Abstract

Infections caused by human immunodeficiency virus (HIV) are considered one of the main public health problems worldwide. Antiretroviral therapy (ART) is the current modality of treatment for HIV-1 infection. It comprises the combined use of several drugs and can decrease the viral load and increase the CD4^+^ T cell count in patients with HIV-1 infection, thereby proving to be an effective modality. This therapy significantly decreases the rate of morbidity and mortality owing to acquired immunodeficiency syndrome (AIDS) and prolongs and improves the quality of life of infected patients. However, nonadherence to ART may increase viral resistance to antiretroviral drugs and transmission of drug-resistant strains of HIV. Therefore, it is necessary to continue research for compounds with anti-HIV-1 activity, exhibiting a potential for the development of an alternative or complementary therapy to ART with low cost and fewer side effects. Natural products and their derivatives represent an excellent option owing to their therapeutic potential against HIV. Currently, the derivatives of natural products available as anti-HIV-1 agents include zidovudine, an arabinonucleoside derivative of the Caribbean marine sponge (*Tectitethya crypta*), which inhibits the reverse transcriptase of the virus. This was the first antiviral agent approved for treatment of HIV infection. Additionally, bevirimat (isolated from *Syzygium claviflorum*) and calanolide A (isolated from *Calophyllum* sp.) are inhibitors of viral maturation and reverse transcription process, respectively. In the present review, we aimed to describe the wide repertoire of natural compounds exhibiting anti-HIV-1 activity that can be considered for designing new therapeutic strategies to curb the HIV pandemic.

## 1. Introduction

Since the emergence of the human immunodeficiency virus (HIV) and with time, the pandemic caused by this virus has been established as one of the main public health problems worldwide, generating new challenges in terms of its prevention and control [1]. Currently, approximately 38 million people are infected with the virus, of which around 32% are not receiving antiretroviral treatment; 1.7 million individuals were newly infected in 2019, and an average of 690.000 deaths occur each year from acquired immunodeficiency syndrome (AIDS)-related diseases [2].

The complex and successful life cycle of HIV prevents its natural removal from the host mediated by the immune system [3]. The action of viral proteins is essential during the HIV replication cycle; these proteins include reverse transcriptase (RT), which synthesizes double-stranded DNA from single-stranded viral RNA, a fundamental step for viral replication; protease, which processes viral polyproteins and converts them into functional proteins; gp120 and gp41 glycoproteins, which facilitate the fusion of the viral membrane with the host cell membrane; and integrase, which mediates viral genome integration within the host DNA [3, 4].

These proteins are fundamental in the pathogenesis of HIV and represent the main targets of antiretroviral therapy (ART), which generally include three active drugs from two or more drug classes [5]. This is currently considered the standard treatment for HIV infection [6] and has radically changed the prognosis of HIV infection because ART can reduce the viral load, increases the CD4+ T cells count, and reduces the probability of new opportunistic infections, thereby significantly lowering the morbidity and mortality of AIDS and extending the life of infected individuals [7–9]. However, specific issues are associated with the use of ART, including limited access to medications and various side effects associated with their use, such as lipodystrophy and metabolic disturbances associated with protease inhibitors (PIs), hypersensitivity and hepatotoxicity due to nucleoside reverse transcriptase inhibitors (NRTIs), and mitochondrial toxicity related to non-nucleoside reverse transcriptase inhibitors (NNRTIs) [10, 11], which decrease treatment adherence, favoring the emergence of some viral strains resistant to therapy [12–15]. Since 68% of the expenses incurred by the healthcare system for the medical attention of a person with HIV correspond to antiviral drugs [16], research is ongoing for new compounds or molecules capable of significantly inhibiting viral replication—with few or no side effects—that can be developed as therapeutic agents and become easily accessible worldwide [17–19].

Natural products have garnered special interest because of the existing biodiversity of flora worldwide and the ease of obtaining extracts and crude forms from these sources with the help of technological innovation [17]. Therefore, it is important to know the wide repertoire of natural compounds exhibiting anti-HIV activity, considering that their identification is essential for designing new therapeutic strategies to counteract the morbidity and mortality associated with the HIV-1 pandemic.

Owing to the great variety of compounds that may be found, the concentration of drug required for 50% inhibition (IC_50_) and selectivity index (SI) of each compound must be particularly considered. SI indicates the number of times that the IC_50_ must increase to achieve a cytotoxic effect of 50%, which means that the compounds considered having therapeutic potential are those with SI of ≥10 [20].

**Figure 1:**
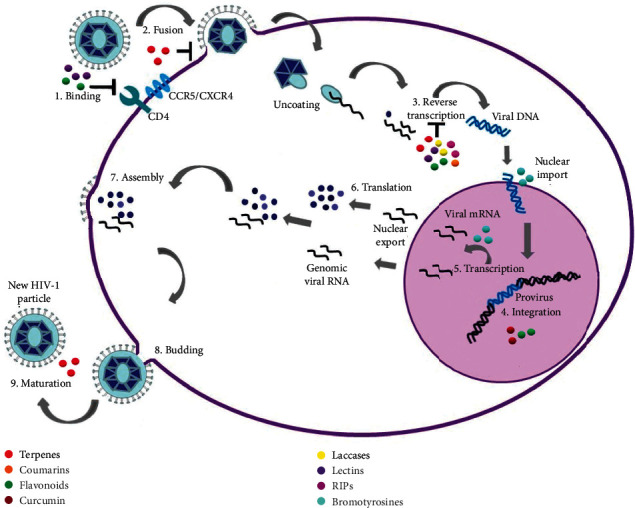
HIV-1 replication cycle exhibiting the sites of action of different natural products with anti-HIV-1 activity. Evidence suggests that flavonoids and lectins have an inhibitory effect on viral binding (1); terpenes inhibit virus fusion (2), whereas laccases, ribosome-inactivating proteins (RIPs), lectins, flavonoids, coumarins, and terpenes act on reverse transcriptase (3). Conversely, flavonoids and curcumin inhibit viral DNA integration (4), and bromotyrosines act on the transcription of the viral DNA (5). Finally, it has been reported that several terpenes act as inhibiting protease-mediated maturation of viral particles (9).

This work aimed to review some of the main natural compounds with anti-HIV activity that have been purified or derived from fungi, plants, and marine sponges and to describe their possible mechanisms of inhibition of HIV-1 replication. Considering that there is a wide range of natural compounds with potential anti-HIV activity, we mainly focused on terpenes, coumarins, flavonoids, laccases, lectins, ribosome-inactivating proteins (RIPs), and bromotyrosines (Figure 1).

## 2. Terpenes

Terpenes are secondary metabolites synthesized by plants, fungi, and animals. They are classified based on the number of isoprene units (C5) present in their chemical structure [21]. Different types of terpenes exhibiting anti-HIV activities include monoterpenes, diterpenes, triterpenes, and triterpene derivatives (e.g., saponins) [22]. The anti-HIV activity of several terpenes has been evaluated in different cell lines, which has led to the identification of metabolites with antiviral effects, thereby demonstrating their potential pharmacological use in the prevention of this infection. They have been isolated from numerous plant species, such as *Cipadessa cinerascens, Trigonostemon* sp*., Anisomeles indica*, and *Gardenia carinata*, as well as from fungal species, such as *Ganoderma lucidum* (Table 1).

**Figure 2:**
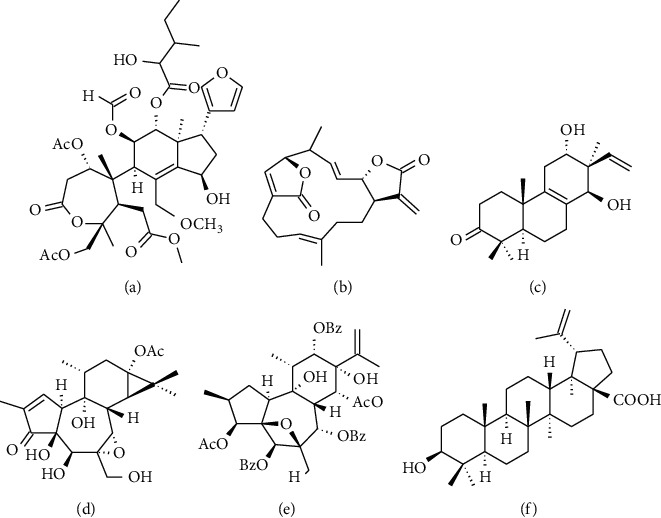
Chemical structure of representative terpenes with antiviral activity against HIV-1. (a) Ciparasin A. (b) Ovatodiolide. (c) Excocarinol A. (d) Excoecafolin A. (e) Trigothysoid J. (f) Betulinic acid.

Monoterpenes and ciparasins B and P (Figure 2(a)), isolated from the cactus *C. cinerascens*, have shown significant anti-HIV activity; a cytotoxicity test using the 3-(4,5-dimethylthiazol-2-yl)-2,5-diphenyltetrazolium bromide (MTT) assay showed that the anti-HIV activity of these metabolites is attributed to the inhibition of the cytopathic effect generated by HIV-1 NL4-3 on MT-4 cells, which have lymphoblast features and are highly susceptible to this viral infection [23].

Regarding diterpenes exhibiting anti-HIV activity, ovatodiolide (Figure 2(b)), isolated from the plant *A. indica*, inhibits 80%–90% of the cytopathic effect of HIV-1 [24].

Conversely, Huang et al. isolated a diterpene from the stems of the plant *Excoecaria acerifolia* Didr., which is designated as excocarinol A (Figure 2(c)) [25]. Subsequently, using the same plant, they reported four tigliane- and daphnane-type diterpenes called excoecafolins A (Figure 2(d)), B, and C and daphnopsis factor R2, respectively. The anti-HIV activities of these compounds were evaluated by determining the inhibition of the cytopathic effect caused by the virus, measured by syncytium formation and moderate cytotoxicity quantified with the MTT assay on the C8166 cell line [26].

The activity of plants belonging to *Trigonostemon* sp. has been studied. Li et al. evaluated the cytopathic effect of the virus in the presence of four daphnane-type derivatives of *Trigonostemon lii*. Two of these were synthesized de novo and named trigolins C and G; the remaining two compounds evaluated have previously been reported as trigochinins A and F. Consequently, these compounds inhibited syncytium formation in the C8166 cell line [27]. Similarly, Cheng et al. isolated four new daphnane-type diterpenoids of *Trigonostemon thyrsoideum* called trigothysoids J (Figure 2(e)), L, N, and O with moderate anti-HIV activity and evaluated four other compounds previously reported as rediocides A, C, and F and trigonosin F, which displayed potent antiviral activity (half-maximal effective concentration (EC_50_) from 0.001 to 0.015nM) [28].

Regarding triterpenes of plants with anti-HIV activity, research was mainly focused on compounds derived from betulinic acid (Figure 2(f)), ursolic acid, and oleanolic acid.

Betulinic acids are pentacyclic triterpenes of natural origin, mainly found in the external bark of various plants that are often used in the timber industry. Particularly, they have been extracted from the methanolic extracts of *Quisqualis fructus*, dichloromethane extracts of *Coussarea paniculata* twigs, and ethanolic extracts of *Anemone raddeana* roots as well as from *Vitex negundo*, *Doliocarpus schottianus* leaves and wood, and *Syzygium claviflorum* leaves, among others [99]. These acids proved to have a wide variety of biological activities, such as antibacterial, antimalarial, anti-inflammatory, antioxidative, anticarcinogenic, and anti-HIV effects. Their anti-HIV activity includes the inhibition of the entry to the target cell and inhibition of viral protease and RT; therefore, they may be used in combination with conventional antiretroviral therapy [99].

Fujioka et al. isolated the betulinic, platanic, and dihydrobetulinic acid components from *S. claviflorum* leaves, which exhibited anti-HIV activity by inhibiting the replication of the HIV-1 IIIB in H9 cells, as measured by detecting p24 antigen using enzyme-linked immunosorbent assay (ELISA), without significantly altering cell growth; this indicated that these compounds have a direct effect on viral replication and that the anti-HIV activity not caused by generalized cell cytotoxicity [29].

Other authors modified betulinic acid by introducing the 3,3-dimethylsuccinyl group replacing the acid C3 hydroxyl, generating 3-O-(3′,3′-dimethylsuccinyl) betulinic acid (bevirimat), resulting in a considerable increase of its anti-HIV activity. Kanamoto et al. evidenced the ability of this compound to inhibit HIV-1IIIB-induced cytopathic effect by MTT assay, further corroborated its anti-HIV activity by inhibition of p24 antigen expression in peripheral blood mononuclear cells (PBMCs) [30]. In conjunction, to determine the viral cycle step affecting bevirimat, they performed syncytium formation inhibition assays with Molt-4 cells, infectious virus release by multinuclear activation of galactosidase indicator (MAGI) assay with MAGI-CCR5 cells, electron microscopic observation, and the time-of-addition assay [30]. The compound did not inhibit the formation of syncytia but inhibited the release of virus from infected cells; likewise, p24 antigen expression was different when it was associated to the cell where a variation of the enzyme concentration was observed according to the time of compound addition, while low levels were observed in the supernatant of treated cells. Therefore, these findings suggest that bevirimat interferes with HIV-1 assembly or exit [30]. In a subsequent study on this compound using MT-2 cells and PBMCs infected with HIV-1 NL4-3, a decrease in the p24 antigen levels was observed, indicating the inhibition of viral replication [31]. Similarly, electronic microscopy using HeLa cells transfected with HIV demonstrated that modification of this compound is responsible for the inhibition of virus maturation, interfering with the standard processing of the polyprotein encoded by *gag* gene and leads to the production of defective capsids, resulting in a noninfectious virus [31].

The compound bevirimat was one of the first members of a candidate class of drugs against HIV and was tested in phase I (*n* = 32) [100], *n* = 42) [101] and II clinical trials (*n* = 24) [102]. Although global results of this clinical trial were positive, showing efficacy and safety [100, 101], the subsequent phases of the trial were not conducted owing to an ensuing description of a reduction in the efficacy of this drug in patients who were hosts of an HIV strain with mutations in a *gag* gene region, conferring a natural resistance to the drug [103]. Currently, trials with compounds derived from bevirimat are conducted, which undergo structure modifications to potentiate their antiviral activity [104]. Researchers, such as Zhao et al., have evaluated this compound by incorporating structures, such as caffeic acid and piperazine, which potentiated the activity against HIV-1 NL4-3 and bevirimat-resistant HIV strains (such as V370A), thereby inhibiting viral replication in MT-4 cells, as assessed by measuring p24 antigen levels with ELISA and virus maturation in TZM-bl cells [32].

In a structure-activity relationship (SAR) study, the triterpene RPR103611, derived from betulinic acid, was found to interfere with the fusion of HIV-1 with the cell for subsequent entry [33]. Furthermore, it was proposed that its antiviral activity depended on the stability of the envelope complex gp120/gp41, considering that this compound stops the fusion of CD4+ T cells with infected cells expressing these proteins, indicating the effect on virus entry [105]. However, its therapeutic value is limited owing to the lack of activity on R5 HIV-1 strains exhibited by RPR103611 [106].

Other researchers, such as Tang et al., have identified a new class of betulin-derived *α*-keto-amides, which inhibited HIV-1 maturation in PBMCs. The compound that exhibited anti-HIV-1 activity was GSK8999; it exhibited the activity via the inhibition of gag proteolysis for the separation of the capsid-spacer peptide 1, which is an essential step in virion maturation. This compound was evaluated against a panel of 62 HIV-1 strains and displayed effectiveness against 57 of them. Particularly, activity was observed against a virus with *gag* polymorphism in positions Q369H, V370A, and T371A, which produces resistance to maturation inhibitors, as well as against a wild strain [34]. These results reveal the therapeutic potential of this compound.

Furthermore, it was determined that ursolic acid, isolated from the methanolic extract from the whole plant of *Geum japonicum*, inhibits the activity of the viral protease. To this end, a synthetic peptide containing the cleavage site for p24–p17 was used as the gag precursor, and this cleavage product was quantified using HPLC [35]. Additionally, Kashiwada et al. isolated this compound from the plants *Prunus glandulosa, Phoradendron juniperinum, S. claviflorum*, and *Hyptis capitata*, and they modified it to generate 3-O-diglyoryl-ursolic acid, which inhibited viral replication in H9 cells [36].

Similarly, triterpenes called carinatins A, B, E, F, and G and dikamaliartane D—derivatives from *G. carinata*—displayed anti-HIV activity by reducing syncytium formation in 1A2 cell lines infected with the ΔTat/revMC99 virus, as evaluated by the MTT assay. Additionally, an assessment of the RT activity revealed an effect on this enzyme, particularly by dikamaliartane D [37].

Some triterpenes with anti-HIV activity have been fungal isolates, such as *G. lucidum*. Triterpenic compounds isolated from the fruiting body of this fungus include ganoderic acid H, ganoderiol A and B, ganoderic acid Cl, ganoderic acid B, and ganolucidic acid A, which were assessed using the MT-4 cell line by observing the inhibition of viral replication and moderate activity against the HIV-1 protease. Similarly, the compounds ganoderiol F and ganodermanontriol inhibited the cytopathic effect caused by the virus at a concentration quite lower than that of the previous compounds [38]. Using a cell-free assay, Min et al. evaluated the anti-HIV activity of some of these compounds, quantifying the fragments obtained from the cleavage of a peptide on which the viral protease specifically acted in the presence of these compounds, as assessed by HPLC via the determination of the significant inhibitory activity against the HIV-1 protease [39].

Saponins include the compounds gleditsia saponin C and gymnocladus saponin G, isolated from the fruit of plants *Gleditsia japonica* and *Gymnocladus chinensis*, respectively. They exhibited inhibitory effects against the replication of the HIV-1 IIIB strain in H9 cells, as determined by measuring the p24 antigen levels using ELISA [40].

Therefore, terpenes are important owing to their potential as intermediaries in the synthesis of compounds with anti-HIV activity.

## 3. Coumarins

Coumarins are a wide group of phenolic compounds mainly found in the fruits and flowers of plants and play a role in plant growth, respiration and photosynthesis control, and defense against infections [107].

Coumarins have different biological properties in different cell systems. They have been known to exhibit anti-inflammatory, antioxidative, antiallergic, and anticarcinogenic activities, as well as hepatoprotective effects [107]. Additionally, coumarins and their related compounds structurally isolated from the plants *Calophyllum* sp., *Ferula sumbul*, and *Marila pluricostata* have inhibited HIV replication, thereby presenting strong therapeutic potential (Table 1).

**Figure 3:**
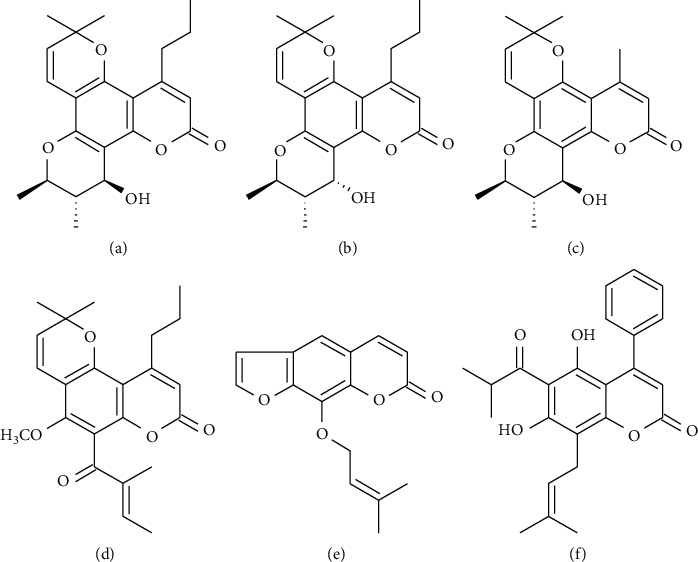
Chemical structure of representative coumarins with antiviral activity against HIV-1. (a) (+)-Calanolide A. (b) (−)-Calanolide B. (c) Cordatolide A. (d) GUT-70. (e) Imperatorin. (f) Mesuol.

Coumarin calanolides A and B, isolated from different species of the rainforest tree *Calophyllum* sp., are a new group of non-nucleoside RT inhibitors (NNRTIs). These compounds were initially isolated from *Calophyllum lanigerum* and evaluated in the CEM-SS cells, and they showed anti-HIV-1 activity via the inhibition of HIV-1 replication [41]. Subsequently, Huerta et al. isolated the compounds (+)-calanolide A (Figure 3(a)) and (−)-calanolide B (Figure 3(b)) from the leaves of *C. brasiliense*. Evaluation of their antiviral activity in MT-2 cells showed that they inhibited the replication of the HIV-1 IIIB strain and RT [42], but due to the low availability of the natural source from which this compound is isolated by the destruction of the natural habitat, the complete synthesis of calanolide A and B became necessary to continue with the pharmacological investigations as a possible antiviral [108–110].

Other researchers have evaluated the activity of the enantiomers of these compounds, observing in vitro activity only in unmodified (+)-calanolide A and (−)-calanolide B (costatolide) with an EC_50_ value of 0.2 and 0.3 *μ*M, respectively [111]. Their effects are attributed to the interaction of these compounds with RT, altering its structural conformation and catalytic complex [111–113]. In particular, (+)-calanolide A has widely been studied owing to its inhibitory activity against zidovudine-resistant HIV-1 strains [41] and its synergic activity in combination with nucleoside analog RT inhibitors and NNRTIs [112].

Additionally, (+)-calanolide A produces cytotoxicity at concentrations 100–200 times higher than the IC_50_ in CEM-SS cells, U937 cells, AA5 cells, PBMCs, and macrophages [113]. Similarly, it has been demonstrated that it produces no toxicity in the hepatic or splenic tissue of mice treated with high doses of this compound [114]. Based on available evidence, phase I clinical trials were initiated on (+)-calanolide A in which the evaluation of its effect in healthy controls showed a favorable pharmacokinetic profile when applying a single dose (*n* = 47) [115] or multiple doses (*n* = 39) in growing concentrations of this compound [116], displaying minimum toxicity, wherein the most common adverse effects were headaches, dizziness, and nausea [115, 116]. These findings suggest that as a novel drug, this compound has high therapeutic activity.

Patil et al. evaluated the antiviral activity of inophyllums B and P, a type of coumarin isolated from the tree *C. inophyllum* Linn., by conducting a scintillation proximity assay, which determines the activity of polymerase-type enzymes; it was found that these compounds inhibited HIV-1 RT. Furthermore, they were active in the culture of Molt-4 cells infected with HIV-1, and a weak cytotoxic effect was observed, as quantified using 2,3-bis-(2-methoxy-4-nitro-5-sulfophenyl)-2H-tetrazolium-5-carboxanilide inner salt (XTT) assay [43].

Cordatolides are another example of an RT inhibitor, which are a type of pyranocoumarin derived from the plant *C. cordato-oblongum* Thw. Dharnaratne et al. found that cordatolide A (Figure 3(c)) and B inhibited RT activity, as assessed in cell-free assays [44] and human osteosarcoma cell systems expressing green fluorescent protein [45].

Another evaluated coumarin is GUT-70 (Figure 3(d)). This compound is a natural product derived from the stem bark of *C. brasiliense*. It has antiproliferative and proapoptotic activities for leukemia and lymphoma [46, 117]. Kudo et al. concluded that GUT-70 inhibits HIV-1 replication in acutely and chronically infected cells. Initially, by measuring the intracellular p24 antigen expression using flow cytometry, GUT-70 was determined to have a dose-dependent capacity to suppress viral replication in U1 cells stimulated with PMA or TNF-*α* as well as to inhibit HIV-1 NL4-3 replication in Molt-4 cells. Subsequently, real-time PCR analysis demonstrated that this compound inhibited HIV-1 Tat-Rev transcription (*p* < 0.05). Furthermore, these investigators analyzed the cytosolic and nuclear NF-*κ*B expression by western blot in U1 cells in the presence or absence of the tested compound and observed that GUT-70 suppressed NF-*κ*B p65 nuclear translocation but not its cytosolic degradation. Additionally, they conducted an electrophoretic mobility shift assay showing suppression of NF-*κ*B binding. Therefore, they concluded that HIV-1 replication was inhibited owing to the suppression of NF-*κ*B [46]. NF-*κ*B is a transcription factor involved in several biological processes, including immunity, inflammation, cancer, and viral infection. Specifically, the NF-*κ*B pathway plays a key role in HIV-1 replication and reactivation of HIV-1 latency [118]. In 2014, Matsuda et al. used GUT-70 to evaluate cell membrane fluidity, an important factor for virus entry, and found a decrease in fluidity using fluorescent depolarization immunoassay in Molt-4 and PM1-CCR5 cell lines. Thereafter, using a fusion model system, it was observed that fused cells decreased in a dose-dependent manner with GUT-70 treatment, concluding that the compound inhibited cell-cell fusion. Subsequently, they found that intracellular expression and p24 antigen production, measured using ELISA, were reduced with GUT-70 treatment in a dose-dependent manner [117]. Recent advances in the development of drugs that inhibit NF-*κ*B activation and their potential applications in inflammatory, autoimmune diseases and cancer are being considered [119, 120]. Although GUT-70 does not act directly against HIV-1, its dual action in inhibiting HIV-1 entry and suppressing NF-*κ*B may be a promising therapeutic strategy against HIV-1.

Imperatorin (Figure 3(e)) is a furanocoumarin isolated from the plant *F. sumbul*. Zhou et al. found that this compound inhibits HIV-1 replication in H9 cells and the growth of uninfected H9 cells [47]. Furthermore, in 2004, Sancho et al. isolated this compound from *Opopanax chironium* and assessed its activity in MT-2 cells infected with pNL4-3-Luc HIV-1 pseudotyped with VSV envelope; they observed a dose-dependent inhibition of the luciferase activity. Moreover, they analyzed the effects in terms of cellular toxicity by employing propidium iodide in HeLa cells, Jurkat cells, MT-2 cells, and PBMCs and observed a cytotoxic effect at concentrations 4–5 times higher than the IC_50_. The mechanism of action of this compound is based on the inhibition of the transcriptional activity of the HIV-1 long terminal repeat (LTR) promoter via a signaling pathway, implying the activation of the Sp1 transcription factor, which interacts with Tat to regulate the activity of this promoter. Imperatorin inhibits Sp1 and inhibits cyclin D1 expression, which stops the cell cycle at phase G1 (phase regulated by Sp1) [121]. On the other hand, in addition to the antiviral activity reported for this compound, it has been described as anti-inflammatory, anticancer, neuroprotective, antihypertensive, and antibacterial activity [122, 123].

Other types of coumarin, such as mesuol (Figure 3(f)) and isomesuol, were isolated from *M. pluricostata*. The antiviral activity of these compounds has been evaluated in Jurkat cells infected with HIV-1 pseudotyped with VSV envelope in which the suppression of HIV-1 replication was observed. Similarly, mesuol demonstrated strong anti-HIV activity in acutely infected PBMCs and inhibited the transcriptional activity of HIV-1 LTR induced by TNF-*α* by interfering with the NF-*κ*B pathway. Additionally, this compound prevented the phosphorylation and transcriptional activity of subunit p65 of NF-*κ*B protein in cells stimulated with TNF-*α* [48].

## 4. Simple and Related Phenolic Compounds

### 4.1. Flavonoids

Flavonoids are polyphenols widely distributed in plants, which favor flower pigmentation and defense against microorganisms and arthropods. They are commonly found in fruits, vegetables, nuts, seeds, stems, and flowers and are typically included in the human diet in large amounts, thereby inhibiting parasitic growth caused by the interruption of some of their metabolic pathways and the decrease of adherence capacity to intestinal cells [124]. For centuries, preparations containing these compounds as the main active ingredients have been used to treat different human illnesses. In vitro studies have shown autoinflammatory, antioxidative, antimicrobial, anticarcinogenic, and antiallergic activities by flavonoids [125].

Flavonoids presenting activity against HIV-1 include derivatives from *Humulus lupulus, Thalassia testudinum*, and *Carmela sinensis*, among others (Table 1).

**Figure 4:**
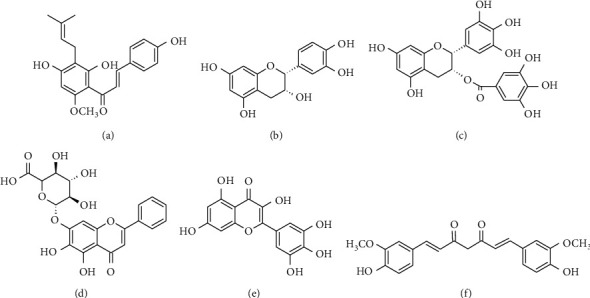
Chemical structure of representative simple and related phenolic compounds with antiviral activity against HIV-1. (a) Xanthohumol. (b) (−)-Epicatechin. (c) (−)-Epigallocatechin. (d) Baicalin. (e) Myricetin. (f) Curcumin.

Some flavonoids, such as xanthohumol and thalassiolin A, exhibit anti-HIV activity owing to the inhibition of different key enzymes involved in viral replication [49, 50]. Xanthohumol (Figure 4(a)), purified from the hops *H. lupulus* used in the production of beer, has shown anti-HIV-1 activity. The anti-HIV-1 activity of this compound was evaluated in C8166 cells, where it demonstrated inhibition of the virus-induced cytopathic effect, inhibition of p24 antigen production, and RT activity, although without the inhibition of virus entry, at noncytotoxic concentrations. This compound further inhibited HIV-1 replication in HIV-1_IIIB_-infected PBMCs [49]. The mechanism of inhibition of this compound was not completely explained in that study, and although it is proposed that the inhibition may occur during the process of reverse transcription, this remains unknown. However, the main mechanism of action for some types of cancer has been described to be based on the detention of growth and apoptosis of the implied cells via the NF-*κ*B pathway [126]. Lastly, a toxicological study conducted on female Swiss nu/nu mice administered xanthohumol (5 × 10^−4^ M) for 4 weeks showed safety when administered orally; no alteration of major organ functions was observed [127]. These results, added to the in vitro antiviral activity observed, indicate that this compound may represent a new therapeutic agent for HIV-1 infection.

Thalassiolin A is a natural flavone isolated from the Caribbean seagrass *T. testudinum*. The effects of this compound on HIV-1 integrase were assessed in the MT-2 cells in which inhibition of the viral cDNA end cutting and its strand transfer to Mg^2+^-dependent cellular DNA were observed. Similarly, thalassiolin A inhibited viral replication in the MAGI cell line and no resistant viral strains were observed, despite treatment for long periods. Additionally, no cytotoxicity was observed in MT-2 and HCT-116 cells [50].

In 1993, Mahmood et al. evaluated a series of flavonoids to determine their anti-HIV potential: (−)-epicatechin (Figure 4(b)) and (−)-epicatechin-3-O-gallate extracted from the plant *Detarium microcarpum*. These compounds were evaluated in the C8166 cell line, showing inhibition of gp120 production, as quantified using ELISA and a 50% cytotoxic concentration, i.e., CC_50_ > 100 *μ*g/mL for both compounds observed in the MTT assay. Additionally, it was determined that (−)-epicatechin-3-O-gallate inhibited syncytium formation in >90% of chronically infected H9 cells. This effect is achieved owing to the irreversible interaction of these flavonoids with gp120, thereby avoiding the fusion of the virus with the receptor CD4 [51]. Green tea (*Camellia sinensis*) contains various catechins and some isomers, such as epigallocatechin gallate (EGCG) (Figure 4(c)). It has been demonstrated that EGCG is one of the most powerful catechins, considering that it inhibits HIV-1 ineffectiveness by interfering in the fusion of gp120 with CD4 at physiological concentrations (6 *μ*mol/L) [128–130]. Moreover, Yamaguchi et al. showed that EGCG is involved in the destruction of viral particles in cells acutely and chronically infected with HIV-1, exerting a dose-dependent effect [128]. Furthermore, in 2011, Li et al. reported the efficacy of this compound as an allosteric inhibitor of HIV-1 RT using HeLa-CD4-LTR-*β*-gal cells infected with HIV-1 [52]. In a phase I study in healthy volunteers, (−)-epicatechin was found to be well tolerated and to cause no adverse effects when administered orally at a single dose (*n* = 9) of 50, 100, or 200 mg or multiple doses (*n* = 8) of 50 mg daily [52]. These results suggest the involvement of flavonoids in the inhibition of different steps of the HIV replication cycle; they also represent a great potential for therapeutic use.

Baicalin (5,6,7-trihydroxyflavone-7-O-*β*-D-glucopyranosiduronic acid) (Figure 4(d)) is a flavonoid derived from the root of *Scutellariae radix*, which displayed the inhibition of HIV-1 replication in PBMCs of healthy and seropositive controls for HIV-1. Antiviral activity was determined using the quantification of the p24 antigen levels with ELISA by observing inhibition of viral replication at dose-dependent concentrations of baicalin, with 72% reduction of the p24 antigen levels and an IC_50_ of 0.5 *μ*g/mL in PBMCs of uninfected individuals, whereas an IC_50_ of 0.2 *μ*g/mL was observed in infected individuals [53]. Additionally, at a concentration of 2 *μ*g/mL, this compound inhibited the activity of HIV-1 RT by 50% in an in vitro assay [53]. Similarly, Li et al. proved that baicalin isolated from *Scutellaria baicalensis* Georgi exhibits antiviral activity by inhibiting viral fusion mediated by the HIV-1 envelope proteins, for the X4 and R5 strains, in cells expressing CXCR4 or CCR5, thereby blocking virus entry [54].

Strong inhibitory activity against HIV-1 RT has been detected with myricetin (Figure 4(e)), which is derived from various plants, such as *Dioscorea bulbifera* and *Marcetia taxifolia* [55–57]. In 2014, Pasetto et al. conducted cytotoxicity tests by the resazurin fluorometric method using TZM-bl cells, HeLa cells, PBMCs, and H9 cells and found cellular viability of ≥85% when myricetin was used at a concentration of 100 *μ*M. Additionally, the same cells were employed to evaluate anti-HIV-1 activity via quantification of the p24 antigen. These cells were infected with strains of HIV-1, such as BaL (tropic R5), MN (tropic X4), and 89.6 (dual tropism X4R5); there was 87% inhibition of HIV-1 BaL in TZM-bl cells, 85% inhibition of HIV-1 MN, and 88% inhibition of HIV-1 89.6 in H9 cells. Similarly, in PBMCs, this compound inhibited HIV-1 MN and HIV-1 89.6 by 86% and 85%, respectively. Finally, it was found that these effects are caused by the inhibition of viral RT [55]. Thereafter, using the multiple-well plate integration assay, Chaniad et al. determined that myricetin exhibited activity against HIV-1 integrase. Furthermore, using a molecular docking technique, it was observed that this compound was located extremely close to the integrase catalytic site and that it interacted with several amino acid residues involved in the 3′-processing and transfer reactions of the integrase strand [56]. Moreover, Ortega et al. assessed the antiviral effect of myricetin; they initially observed inhibition in HIV-1 replication when quantifying the p24 antigen levels using ELISA in MT-4 cells infected with HIV-1. Furthermore, they reported RT inhibition, which was measured with fluorescence assay [57].

These findings reveal the participation of flavonoids in the inhibition of different steps of the HIV-1 replication cycle, mainly the early stages, and demonstrate that they represent a group of substances with high potential for therapeutic use.

### 4.2. Curcumin

Curcumin (Figure 4(f)), a biologically active polyphenolic compound found in the yellow pigment of the plant *C. longa*, is widely used as a spice, food colorant, and preservative and exhibits anti-inflammatory, antioxidative, and anticarcinogenic activities [131]. This compound has exhibited activity against HIV-1 via different mechanisms. First, its activity was assessed in PBMCs infected with HTLV-III_B_ (virus was obtained from the culture supernatant of chronically infected H9 cells) and treated with curcumin, wherein p24 antigen production was reduced in a dose-dependent manner [132]. Thereafter, it was demonstrated that curcumin acts on HIV-1 integrase by interacting with its catalytic center, affecting 3′-processing and strand transfer, thus preventing the viral DNA insertion into the infected cell genome [58]. Furthermore, curcumin degrades Tat, as demonstrated by a transfected HEK-293T cell assay, wherein Tat levels were quantified in the presence of a protein synthesis inhibitor (cycloheximide) with or without curcumin and an increase in Tat degradation rate in the presence of curcumin was observed; however, a semiquantitative analysis using RT-PCR showed that Tat degradation occurred regardless of gene transcription. Additionally, curcumin decreased the Tat-mediated transactivation of the LTR promoter and inhibited the production of the virus, as observed in TZM-bl cells infected by HIV-1, thereby showing up to a 30% decrease in p24 antigen levels [133]. Other curcumin analogs, such as dicaffeoylmethane and rosmarinic acid, inhibit integrase activity in 3′-end processing and transfer of the viral DNA strand to cellular DNA, which was evidenced with the use of short oligonucleotides similar to the virus LTR [59].

Finally, in 2015, Kumari et al. synthesized curcumin A, a new curcumin-derived compound. They initially compared the inhibitory effects of curcumin and curcumin A on strains of HIV-1 luciferase (Luc) generated by a single round of infection in CEM-T cells and found similar IC_50_ values. Furthermore, in PBMCs, curcumin A showed a stronger inhibition of HIV-1 compared with curcumin. Furthermore, these compounds reduced the viability of CEM-T cells and PBMCs, which was quantified using calcein-AM and trypan blue, respectively. Using real-time PCR, they reported a reduction in the expression of gag and env mRNA encoded by this virus in cells treated with these compounds. Moreover, they quantified the early and late products of viral reverse transcription and found that curcumin and curcumin A showed similar inhibition of early transcription, whereas only curcumin A exhibited the inhibition of late transcription. Therefore, this modification renders curcumin A more efficient by inhibiting both stages of transcription, thus favoring its antiretroviral activity [60].

Curcumin has proven to be a safe compound when used in mice and humans, even when orally administered at high doses (8000 mg/day) to non-HIV population for 3 months [134]. However, it is not well absorbed when administered via this route. Therefore, a study was conducted combining curcumin with hepatic and intestinal metabolism inhibitors, and an increase in its bioavailability was observed [135].

Certain curcumin analogs, such as L-chicoric acid (L-CA), showed an inhibitory effect on integrase and HIV-1 entry in MT-2 and H9 cell lines, respectively. A disintegration assay was conducted to determine this using a DNA probe connected to L-CA, and it was observed that integrase acts in an unspecific manner by cutting viral DNA. Using reaction kinetics assays, the noncovalent interaction between L-CA and viral integrase was determined. Therefore, it can be concluded that L-CA is a noncompetitive but reversible inhibitor of HIV-1 integrase [61].

## 5. Proteins

Among the great diversity of existing proteins, three main types of proteins exhibiting anti-HIV-1 activity have been identified: laccases, lectins, and RIPs.

### 5.1. Laccases

Laccases are a group of enzymes that belong to the polyphenol oxidase group and catalyze the reduction of oxygen to water via the oxidation of phenolic substrates [136]. They are used in textile dyes, biosensors, and contaminated water detoxification, among other biotechnological processes, in addition to participation in lignin polymer formation in plants and lignin degradation as well as in pathogenic processes in fungi [69]. The latter are mainly included in the phylum Basidiomycota, which participates in wood decomposition [136].

Recently, certain fungal laccases have exhibited anti-HIV-1 activity, among other properties, gaining the interest of the scientific community [69, 136, 137]. Researchers such as Wang and Ng have published a series of investigations, wherein they have isolated, purified, and physically and chemically characterized fungal laccases and have evaluated their capacity to inhibit HIV-1 infection via an action against virus RT in free-cell assays. These investigators observed anti-HIV activity in laccases isolated from the fruiting bodies or mycelia of different fungal species shown in Table 1, with an IC_50_ range of 0.06–22 *μ*M [62–74].

Laccases isolated from *Hericium coralloides* [62], *Tricholoma mongolicum* [64], *G. lucidum* [65], and *Agaricus placomyces* [66] exhibit the lowest IC_50_, allowing their use in the development of antiretroviral medications. However, it must be noted that the obtained IC_50_ values are not absolute indicators of a compound's potential and that SI must be considered to understand the potential effectiveness of the medication at the pharmacological level [67]. These researchers did not include a SI value in their publications, and their results are based on cell-free in vitro assays using which RT activity is measured; therefore, it is important to study anti-HIV activities of different laccases at the cellular level and to evaluate the inhibition of this enzyme and other viral proteins.

The molecular mechanisms involved in the inhibitory effect of laccases remain unclear. However, researchers Wang and Ng suggest that there is a protein-protein interaction between these enzymes and RT [65, 67, 68], as observed by Böttcher and Grosse in 1997 [138]. Using immunoprecipitation/coprecipitation and western blot assays employing antibodies against these enzymes, these researchers concluded that completely processed RT inhibition may be owing to direct interaction with the protease of the same virus [138]. Some laccases, such as those extracted from the fungal species *Cantharellus cibarius* [139] and *Albatrella dispansus* [140], lack this ability. This is because these enzymes vary at the inter- and intraspecies levels and even in terms of the isoenzymes produced in different tissues in the same organism. This is justified by the presence of multiple genes in fungi that encode for the enzyme, and enzymes may vary in their percentage of glycosylation, generating interference in the interaction between the enzyme and viral proteins [136].

Zhao et al. reported that mycelia of the fungus *Coprinus comatus* contain an enzyme that is a strong suppressor of HepG2 (hepatocellular carcinoma) and MCF7 (human breast adenocarcinoma cell line) cell line proliferation and an inhibitor of HIV-1 RT, as assessed using ELISA, indicating that it is an antipathogenic protein [69]. Similarly, from the fungus *Pleurotus cornucopiae*, a new laccase with a molecular mass of 67 kDa was isolated from fermentation broth via ionic exchange chromatography and gel filtration [70, 71]. This laccase inhibits HIV-1 RT, and this activity was considerably inhibited by Fe^3+^ and Hg^2+^ and stimulated by Cu^2+^ and Pb^2+^ [70].

Laccase-producing basidiomycete fungi are distributed throughout the world. For example, in Colombia, to date, there are four genera of laccase-producing fungi attributed with anti-HIV activity reported in the local microflora, such as *Clitocybe* sp., *Tricholoma* sp. [141], *Lentinus* sp. [142], and *Ganoderma* sp. [143]. Within the vast unexplored flora inside and outside Colombia, there may be several other genera with therapeutic potential for HIV infection. Therefore, at the international and local levels, this wide group of laccases offers a global alternative for studying new compounds with anti-HIV activity with a potential for the development of antiretroviral therapies. Prior studies conducted by our research group show that laccases generate an inhibitory effect on HIV-1 replication and the viral reverse transcription process, which are evaluated in terms of early and late transcribed levels [144]. The maximum percentage inhibition of HIV replication was 86.4% and was obtained using an extract enriched with laccase derived from the fungus *Lentinus* sp., as measured with flow cytometry for green fluorescent protein (GFP) in U373-MAGI cells infected with a construct (pNL4-3 delta env) with a VSV envelope and marked with GFP. Additionally, the maximum percentage of inhibition of replication measured using ELISA for the p24 antigen was 68.1% and 60.1% for the extract enriched with laccase from *Ganoderma* sp. and *Lentinus* sp., respectively. Similarly, the percentages of maximum inhibition for the early transcripts were 86.6% for *Ganoderma* sp. and 91.3% for *Lentinus* sp. Finally, the percentages of maximum inhibition of late transcripts were 93.6% for *Ganoderma* sp. and 90.5% for *Lentinus* sp. The laccase produced by these fungi appeared to be more efficient than those of other fungi in inhibiting the reverse transcription process [144]. These results provide the basis for future research, wherein the mechanism of action of the purified enzyme of these fungi, their biochemical properties, and other potential applications in pharmacology should be explored. To date, there are no reports on laccases from a nonfungal source with anti-HIV-1 activity.

### 5.2. Lectins

Lectins are di- or polyvalent carbohydrate-binding proteins and are also known as hemagglutinins owing to their ability to agglutinate cells of the human immune system, regardless of whether they originate from a fungal or vegetable source [145]. Lectins are distributed in diverse living organisms, including animals, plants, bacteria, viruses, and fungi [146]; in the latter, they are involved in latency, growth, morphogenesis, and morphological changes following parasite infection as well as in molecular recognition during the early stages of fungal mycorrhization [147].

These proteins have garnered particular scientific interest because they display a series of exploitable biological activities, including antitumoral, antiproliferative, mitogenic, and immunopotentiation effects [84, 86, 148–151]. Additionally, the anti-HIV-1 activity demonstrated by lectins isolated from several fungal species is of special interest in the present study (Table 1) [75–87]. Although the mechanism of action by which these lectins inhibit RT activity remains unknown, researchers have suggested that it is owing to protein-protein interaction [84, 86, 87].

According to studies, lectins may be found in the fruiting bodies and mycelia of certain fungi. Similarly, it must be considered that the content of this compound varies based on age and fungal recollection time. Moreover, some fungal species have several lectins that may be closely related, differing in their isoelectric points; lectins isolated from the common mushroom *Agaricus bisporus* are an example [152]. These proteins may also vary in their molecular weight owing to their subunits' amino acid changes, as can be observed in lectins isolated from the mushroom *P. cornucopiae* [153]. Wang et al. mentioned that by 1998, 100 lectins were isolated from fungi, plants, and microorganisms [146]. A more detailed analysis of different lectin variants found in each of the specimens used to date may facilitate establishing a greater and comprehensive database of the types of lectins that inhibit HIV-1 RT. In particular, if tests are conducted to evidence other targets of virus inhibition besides RT, the number of compounds with therapeutic potential would significantly increase.

Other researchers have observed that a series of mannose-specific agglutinins (lectins) derived from the plants *Galanthus nivalis, Hippeastrum hybrid, Narcissus pseudonarcissus, Listera ovata, Cymbidium hybrid*, and *Epipactis helleborine* inhibit the cytopathic effect of HIV-1 on MT-4 cells [76, 77].

Other lectins present in plants, algae, and marine invertebrate animals exhibit anti-HIV activity [154]. *Myrianthus holstii* lectin, derived from the tree *M. holstii*, was assessed in CEM-SS cells; this lectin was found to be protective against the cytopathic effects of HIV-1 (syncytium formation) in nontoxic concentrations for the cell, suggesting that this lectin reversibly inhibits HIV-1 infection [78]. *Oscillatoria agardhii* agglutinins (OAAs) are generated by the green algae *O. agardhii*. Férir et al. reported that OAAs exhibited anti-HIV-1 activity in MT-4 cells; the researchers also used PBMCs and showed that OAAs inhibit X4, R5, and X4/R5 viral strains and inhibit several M-group HIV-1 subtypes. To prove that the effects mentioned were not the result of a cytotoxic effect on cells, the MTS/phenazine ethosulfate method was implemented in PBMCs and MT-4 cells for evaluating cell viability. Similarly, they described that this lectin inhibited syncytium formation in infected and uninfected CD4+ T cells in a dose-dependent manner [79].

Swanson et al. reported that the BanLec lectin, isolated from *Musa acuminate* banana, exhibits anti-HIV-1 activity. Therefore, ELISA was performed, which showed that the activity of this compound is the result of direct binding to gp120, a protein found in the viral envelope, thereby preventing virus entry into the target cell [155].

Griffithsin (GRFT) is derived from red algae *Griffithsia* sp., which showed potent activity against several enveloped viruses. This compound inhibited the HIV replication in CEM-SS cells and PBMCs infected with HIV-1 isolates (R5 and X4 strains) in which the RT activity and p24 antigen levels decreased and a blockade of cell-to-cell fusion and transmission of HIV-1 infection were observed [156]. This compound is one of the most powerful inhibitors of HIV entry because it inhibits viral replication at the plasma membrane level and specifically targets multiple mannose-rich residues present in the gp120 of the viral envelope. Therefore, it is proposed to be a promising compound for the potential development of a new antiviral drug [157]. This compound inhibited HIV entry by targets multiple mannose-rich residues present in the gp120 of the viral envelope. Therefore, it is proposed to be a promising compound for the potential development of a new antiviral drug [157]. This compound also blocked infectivity against clades A, B, and C of the HIV [157–159]. Accordingly, studies have shown that GRFT is stable in a wide range of temperatures and pH, stable in the cervicovaginal mucosa of macaque monkeys, demonstrating its potential as a topical microbicide [158].

In studies on human cell lines, Griffithsin showed high safety and efficacy profile because it has no mitogenic activity and does not induce the secretion of proinflammatory cytokines [160]. Subsequently, the effect of this compound was evaluated in two rodent species and minimal toxicity was found generating splenomegaly and hepatomegaly after application of single and repeated daily subcutaneous doses [161]. In addition, GRFT synergizes with the HIV-1 drugs tenofovir, maraviroc, and enfuvirtide, which could be used for microbicide development [162]. Kramzer et al. performed in vitro characterization of a preformulation of this compound and found physical, chemical, and biological stability profiles under stress conditions, except oxidative stress [163]. GRFT, being a lectin, is prone to oxidation; to overcome this property, an oxidation-resistant form of GRFT (Q-GRFT) has been developed and evaluated in rhesus macaque rectal tissue samples showing a nontoxic effect [164]. Also, new delivery strategies with different vehicles are being explored to improve its bioavailability [165, 166].

To date, two phase-I clinical studies have been initiated to investigate the potential toxicity of GRFT in healthy populations. The first study aims to evaluate the safety of GRFT in a vaginally applied gel in healthy women [167]. The second ongoing study is called PREVENT (pre-exposure prevention of viral entry), which evaluates the safety and pharmacokinetics of Q-GRFT enema administered rectally in adults practicing receptive anal intercourse [168].

Similarly, lectins found in *Phaseolus vulgaris*, *Momordica charantia*, and *Ricinus communis* inhibited HIV-1 RT and the N-glycohydrolase enzymes (*α*-glucosidase and *β*-glucosidase) [75]. N-glycohydrolase enzymes are commonly found in the Golgi apparatus of host cells and are responsible for protein glycosylation. Another study showed a decrease in the infectivity of HIV virions caused by the inhibition of these proteins, considering that viral glycoproteins are highly glycosylated [169].

It has been suggested that several lectins preserve their biological activity as they pass through the digestive tract [146]. This implies that the anti-HIV activity of lectins isolated from some fungi, such as *A. bisporus* and *P. vulgaris*, can maintain their antiviral effect in the digestive tract after consumption; therefore, they can be explored for possible benefits as a frequent component of the diet of patients with HIV or of uninfected individuals as a protective measure. This, in addition to the fact that some lectin-producing fungal genera, such as *Boletus* sp., are largely distributed worldwide and particularly in Colombia [141], renders research on lectins an area of interest for HIV researchers.

### 5.3. Ribosome-Inactivating Proteins (RIPs)

RIPs are compounds that irreversibly inhibit ribosomes, which are found in eukaryotic cells. For the inhibitory action, RIP uses its RNA-N-glycosidase activity, which hydrolyses the N-glycosidic bond between the adenine and ribose of the nucleoside A-4324 of the 28S subunit of rRNA. This released adenine can be found in a preserved location essential for ribosomal function in eukaryotes [170]. RIPs are reportedly present in several angiosperms, fungi, and bacteria [171]. They are involved in a series of activities, including the inhibition of translation and N-glycosidase as well as antimitogenic, immunomodulating, antiproliferative, antifungal, and antiviral activities [170]. The antiviral activity includes the inhibitory ability of HIV-1 RT.

The HIV-1 RT inhibitory activity in fungi has been described by Wang and Ng, who reported RIPs as virus inhibitors (Table 1). Velutin, an RIP composed of a single chain with a molecular weight of 13.8 kDa, was isolated from winter mushroom *Flammulina velutipes*. This protein completely inhibited HIV-1 RT at a concentration of 5 mg/mL [88]. Following succinylation, its activity showed an increase because this process led to changes, such as variation in the net load of the protein, which may significantly alter its possible interactions with other molecules, similar to that described for succinylation of ovalbumin [172].

Although other RIPs that act on HIV-1 RT, such as lyophyllin [89], hypsin [90], and marmorine [91], have been identified, little is known about their mechanism of action.

Numerous RIPs have been identified in several plant families, including trichosanthin (TCS) isolated from the Chinese medicinal herb *Trichosanthes kirilowii*, which has immunoregulatory, anticancer, and anti-HIV activities [173]. In 1989, McGrath et al. reported the antiviral activity exerted by this compound when inhibiting HIV-1 replication. This inhibition was observed in acutely infected T cells, which showed a decrease in cytopathic effects and p24 antigen levels. Furthermore, a decrease was reported in the p24 antigen levels of chronically infected macrophages [174]. Subsequently, Wang et al., after conducting an in vitro test using H9 cells, found that the anti-HIV activity of TCS was achieved via the apoptotic effect in infected cells. At a concentration of 24 *μ*g/mL, trichosanthin-induced apoptosis occurred in 8.4% of uninfected H9 cells and 24.5% of H9 cells chronically infected with HIV-1 [175]. Nevertheless, the antiviral mechanism remains controversial because several researchers have reported that this effect is owing to its activity in HIV-1 integration because HIV-infected 293T cells showed a dosage-dependent blocking in HIV-1 integration. Moreover, a glutathione S-transferase pull-down assay was conducted to observe interactions between in vitro proteins, and it was possible to establish that TCS specifically inhibits HIV-1 integration by depurating HIV-1 LTR [176]. Other investigators have shown that TCS can penetrate the viral membrane via lipid rafts, wherein it is protected against protease digestion. Although this internalization does not directly destroy the virus, it can significantly reduce its replication [177].

Patients with AIDS have been included in several clinical trials conducted on TCS. During the first phase I/II clinical test, a decrease was observed in the p24 antigen levels and an increase was observed in the blood CD4+ T cell count [178]. Thereafter, this drug was tested in phase II clinical trials as an adjunct to zidovudine treatment, a classical medication of ART, which resulted in an increase in the CD4+ T cell count during the treatment period [179]. In the same year, Kahn et al. conducted a study including 22 patients with AIDS to evaluate the safety, pharmacokinetics, and immunological effects of multiple doses of this drug. The results showed an increase in the CD4+ and CD8+ T cell count of up to 28 days after the application of the compound [180]. Nonetheless, patients have experienced several side effects, which were probably associated with cytotoxic effects; therefore, it is important to consider different variables related to its use or its chemical structure to increase the antiviral potential of this compound in the future.

Luffin P1 is a small RIP exhibiting anti-HIV-1 activity that is isolated from the seeds of the plant sponge gourd *Luffa cylindrica*. This protein was evaluated in the C8166 cell line in which it inhibited syncytium formation and p24 antigen production. Luffin P1 was cytotoxic in uninfected cells; however, its effect on HIV replication was considerably stronger. In the present study, we demonstrated that luffin P1 interacts with Rev response elements (RRE) in a dose-dependent manner, indicating that it could compete with Rev for RREs in infected cells, thereby decreasing the mRNA export rate of HIV-1 to the cytoplasm for protein synthesis and virion assembly [92].

Balsamin, which is isolated from the bitter melon *Momordica balsamina*, inhibits HIV-1 replication in T cell lines and primary human CD4+ T cells. This antiviral compound has a high likelihood of exerting its activity on the viral translation of proteins before budding and viral release. Also, this RIP prevents the replication of the influenza virus [93].

Zarling et al. reported that the antiviral protein isolated from *Phytolacca americana* leaves, known as pokeweed antiviral protein (PAP), inhibits p24 antigen production, as measured using radioimmunoprecipitation in HIV-infected primary CD4+ T cells and macrophages, with an IC_50_ of 0.5 nM [181]. At a later stage, the same investigators showed that compared with nonconjugated PAP, PAP conjugated with monoclonal antibodies against CD4 (anti-CD4-PAP) showed more efficient inhibition of viral replication in CD4+ T cells infected with HIV-1 [182]. The PAP isomers (PAP-I, PAP-II, and PAP-III) exert anti-HIV-1 activities by inhibiting viral replication (quantified using ELISA with the p24 antigen) in PBMCs [94]. In 2015, Krivdova and Hudak reported that the mRNA of the Vif protein of HIV is the PAP substrate. Vif induces degradation of APOBEC3G, a potent inhibitor of the HIV reverse transcription process; therefore, PAP restores APOBEC3G levels, facilitating its proper functioning as a protective factor against viral infections [183].

## 6. Bromotyrosines

Bromotyrosines are a type of molecule whose chemical structure is based on L-tyrosine, showing replacements by bromide atoms at the third or the third and fifth positions, generating mono- or dihalogenated substances, respectively [184]. They can be found in several animal and plant tissues, being mainly isolated from marine sponges of the order Verongida [185] (Table 1). Currently, these compounds have thoroughly been studied owing to their cytotoxic, antiparasitic, anticancer, and antiviral activities [97]. Several bromotyrosine-derived compounds with anti-HIV-1 activity have been reported.

**Figure 5:**
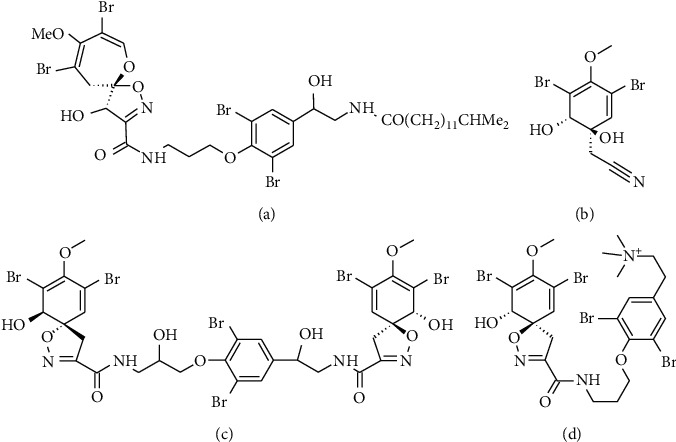
Chemical structure of bromotyrosines with antiviral activity against HIV-1. (a) Psammaplysine D. (b) Purealidin B. (c) Fisturalin-3. (d) Aeroplysinina-1.

In 1993, Ichiba et al. reported that psammaplysine D (Figure 5(a)) exhibits anti-HIV activity by presenting 51% inhibition at 0.1 pg/mL against the HIV-1 RF strain from Haiti [95]. Subsequently, using the RT assay, it was possible to determine that aeroplysinin-1 (Figure 5(b)) and fistularin-3 (Figure 5(c)) isolated from the Caribbean sponge species *Verongula rigida* and *Aiolochroia crassa*, respectively, which are sponges of the order Verongida, inhibited HIV-1 LAI replication in PBMCs [96]. Results of our studies showed that these compounds, along with purealidin B (Figure 5(d)), exhibited anti-HIV-1 activity. To this end, the U373-MAGI cell line infected with a pseudotyped virus (HIV-GFP-VSV-G) in the presence or absence of these compounds and the percentage of cells infected were evaluated using flow cytometry for GFP, and dose-dependent inhibition of viral replication caused by aeroplysine-1 and purealidin B was observed. Similarly, three compounds inhibited reverse transcription and nuclear import of viral DNA as observed via real-time PCR quantification of the early and late transcripts of RT and the viral 2-LTR circle, respectively [97].

Mololipids are another derivative of bromotyrosines that exhibit anti-HIV-1 activity (assessed by conducting a SAR study), which proved to be noncytotoxic to PBMC, as assessed by the MTT assay [98].

## 7. Conclusion

Infections caused by HIV is one of the main public health issues worldwide attributed to the economic, political, and social barriers that limit access to antiretroviral drugs for a large part of the population as well as to the high resistance levels currently reported for the virus. Therefore, research in this area is ongoing even after more than four decades and is aimed at finding a safe treatment that not only inhibits the replication of the virus but also succeeds in restoring the destroyed immune system.

In the present review, we described numerous compounds of natural origin, such as from plants, fungi, and marine sponges, identified as strong inhibitors of various stages of the HIV-1 replication cycle, from entry to transcription, translation, and exit processes. Research on natural products and their derivatives with anti-HIV activity may lead to the development of strong antiretroviral drugs, with few side effects and easy accessibility to patients with HIV infection. This group of molecules includes terpenes, coumarins, flavonoids, laccases, lectins, RIPs, and bromotyrosines as well as their derivatives and agonists. Identification of compounds with the highest therapeutic potential for HIV infection is crucial for the development of future alternative and/or supplemental therapies to ART. Therefore, further research on such compounds is warranted to understand their mechanisms of action against HIV and to conduct clinical trials with the most promising compounds.

## Figures and Tables

**Table 1:** Natural compounds with anti-HIV-1 activity extracted from fungi, plants, or marine sponges.

NC	Source/compound	Origin	Effect	IC_50_/EC_50_^*∗*^	CC_50_	SI/% inhibition ▪	Reference
**Terpenes**	*Cipadessa cinerascens/* a. Ciparasin B	Leaves	Cytopathic effect	a. 5.5 *μ*m/mL^*∗*^	ND	a. >7.2	[23]
	b. Ciparasin P			b. 6.1 *μ*m/mL^*∗*^		b. >6.5	
	*Anisomeles indica/* Ovatodiolide			1.20 *μ*m/mL/0.10 *μ*m/mL^*∗*^	ND	80%–90% **▪**	[24]
	*Excoecaria acerifolia* Didr.*/* Excocarinol A	Stem		>628.9 *μ*M/5.58 *μ*M^*∗*^	ND	>112.7	[25]
	*Excoecaria acerifolia* Didr.*/* a. Excoecafolin A			a. >473.93 *μ*M/0.258 *μ*M^*∗*^	ND	a. >1836.9	[26]
	b. Excoecafolin B			b. 15.52 *μ*M/0.036 *μ*M^*∗*^		b. 431.1	
	c. Excoecafolin C			c. 13.74 *μ*M/0.046 *μ*M^*∗*^		c. 298.7	
	d. Daphnopsis factor R_2_			d. >492.61 *μ*M/0.978 *μ*M^*∗*^		d. >503	
	*Trigonostemon lii/* a. Trigolin C			a. 2.04 *μ*g/mL^*∗*^	a. 54.04	a. 26.49	[27]
	b. Trigolin G			b. 9.17 *μ*g/mL^*∗*^	b. >200	b. >21.81	
	c. Trigochinin A			c. 11.42 *μ*g/mL^*∗*^	c. 106.45	c. 9.32	
	d. Trigochinin F			d. 9.05 *μ*g/mL^*∗*^	d. 86.54	b. 9.56	
	*Trigonostemon thyrsoideum/* a. Trigothysoid J	Twigs and leaves		a. 2.3 ± 1.20 nM^*∗*^	a. >251.2 nM^*∗*^	a. >101.2	[28]
	b. Trigothysoid L			b. 4.0 ± 0.11 nM^*∗*^	b. >296.7 nM^*∗*^	b. >74.0	
	c. Trigothysoid N			c. 0.001 ± 0.76 × 10–3 nM^*∗*^	c. 22.2 ± 0.42 nM^*∗*^	c. 17.619	
	d. Trigothysoid O			d. 0.358 ± 0.50 × 10–2 nM^*∗*^	d. 28.9 ± 5.91 nM^*∗*^	d. 80.8	
	e. Rediocide A			e. 0.004 ± 0.11 × 10–2 nM^*∗*^	e. 22.9 ± 0.64 nM^*∗*^	e. 6077.3	
	f. Rediocide C			f. 0.008 ± 0.98 × 10–3 nM^*∗*^	f. 27.9 ± 0.02 nM^*∗*^	f. 3245.9	
	g. Rediocide F			g. 0.001 ± 0.60 × 10–3 nM^*∗*^	g. 15.2 ± 13.76 nM^*∗*^	g. 15.242	
	h. Trigonosin F			h. 0.015 ± 0.85 × 10–3 nM^*∗*^	h. 23.8 ± 1.49 nM^*∗*^	b. 1618.8	
	*Syzygium claviflorum/* a. Betulinic acid	Leaves	Replication (quantification of p24 antigen)	a. 13 *μ*M/1.4 *μ*M^*∗*^	ND	a. 9.3	[29]
	b. Platanic acid			b. 90 *μ*M/6.5 *μ*M^*∗*^		b. 13	
	c. Dihydrobetulinic acid			c. 13 *μ*M/0.9 *μ*M^*∗*^		c. 14	
	*Syzygium claviflorum/* 3-O-(3′,3′-dimethylsuccinyl) betulinic acid		Cytopathic effect	0.011 *μ*g/mL^*∗*^	14.03 *μ*g/mL	1.275	[30]
			Replication (quantification of p24 antigen)	10.3 nM (VIH WT) and 7.8 nM (drug-resistant HIV-1 isolates)	25 *μ*M	>2.500	[31]
	Derived from betulinic acid	Commercial extract	Maturation	0.015 *μ*M	>4.5 *μ*M	ND	[32]
	3-O-(3′,3′- dimethylsuccinyl) betulinic acid + piperazine and caffeic acid						
	Derived from betulinic acid RPR103611		Fusion	40 ± 19 nM	ND	>100	[33]
	Betulin-derived *α*-keto-GSK8999	Commercial extract	Protease	23 nM (HIV WT), 25 nM (HIV Q369H), 8 nM (HIV V370 A), and 17 nM (HIV T371 A)	ND	ND	[34]
	*Geum japonicum/* Ursolic acid	Whole plant	Protease	ND	ND	85% at 17.9 *μ*g/mL ▪	[35]
	*P*. *glandulosa*, *P*. *juniperinum*, *S*. claviflorum*, H*. *capitata/* 3-0-Diglyoryl-ursolic acid	Leaves	Replication (quantification of p24 antigen)	48.2 *μ*M/0.31 *μ*M^*∗*^	ND	155.5	[36]
	*Gardenia carinata/* a. Carinatin A	Leaves and twigs	Cytopathic effect	a. 27 *μ*M/15.4 *μ*M^*∗*^	ND	a. 1.8/6% ▪	[37]
	b. Carinatin B			b. 72.5 *μ*M/62.7 *μ*M^*∗*^		b. 1.2/1.9% ▪	
	c. Carinatin E			c. 69.5 *μ*M/43.0 *μ*M^*∗*^		c. 1.6/63.7% ▪	
	d. Carinatin F			d. >138.3 *μ*M/41.3 *μ*M^*∗*^		d. >3.4/11.6% ▪	
	e. Carinatin G			e. 46 *μ*M/13.4 *μ*M^*∗*^		e. 3.4/25.5% ▪	
	f. Dikamaliartane D			f. 57.7 *μ*M/˂8.3 *μ*M^*∗*^		f. >7/88.1% ▪	
			RT	f. 68.7 *μ*M			
	*Ganoderma lucidum/* a. Ganoderic acid *α*	Fruiting body	Protease	a. 0.19 mM	g. 15.6 *μ*g/mL h. 15.6 *μ*g/mL i. 15.6 *μ*g/mL	ND	[38]
	b. Ganoderic acid B			b. 0.17 mM			
	c. Ganoderic acid Cl			c. 0.18 mM			
	d. Ganoderic acid H			d. 0.20 mM			
	e. Ganoderiol A			e. 0.23 mM			
	f. Ganoderiol B			f. 0.17 mM			
	g. Ganoderiol F			g. 0.32 mM			
	h. Ganodermanontriol			h. >1 mM			
	i. 3*β*-5*α*-Dihydroxy-6*β*-methoxy ergosta-7,22-diene.			i. 0.18 mM			
	*Ganoderma lucidum/* a. Ganoderic acid *ß*			a. 20 *μ*M	ND	ND	[39]
	b. Lucidumol B			b. 50 *μ*M			
	c. Ganodermanondiol			c. 90 *μ*M			
	d. Ganodermanontriol			d. 70 *μ*M			
	e. Ganolucidic acid A			e. 70 *μ*M			
	*Gleditsia japónica/* Gleditsia saponin C	Fruits	Replication (quantification of p24 antigen)	9.8 *μ*M/1.1 *μ*M^*∗*^	ND	8.9	[40]
	*Gymnocladus chinensis/* Gymnocladus saponin G			14 *μ*M/2.7 *μ*M^*∗*^	ND	5.2	
**Coumarins**	*Calophyllum lanigerum/* a. Calanolide A	Fruit and twigs	RT	a. 20 *μ*M/0.1 *μ*M^*∗*^	ND	a. 200	[41]
	b. Calanolide B			b. 15 *μ*M/0.4 *μ*M^*∗*^		b. 37	
	*Calophyllum brasiliense/* a. (+)-Calanolide A	Leaves	Replication (quantification of p24 antigen)	a. 0.34 *μ*M/mL	ND	ND	[42]
	b. (-)-Calanolide B			b. 0.5 *μ*M/mL			
			RT	ND	ND	a. 81.5% ▪	
						b. 76.2% ▪	
	*Calophyllum inophyllum Linn*.*/*	Leaves and twigs	RT	In a cell-free assay a. 38 nM	a. 55 *μ*M	a. 39 *μ*M	[43]
	a. Inophyllum B			b. 130 nM	b. 25 *μ*M		
	b. Inophyllum P			In Molt-4 cells a. 1.4 *μ*M		b. 16 *μ*M	
				b. 1.6 *μ*M			
	*C*. *cordato-oblongum* Thw*/* a. Cordatolide A	Leaves		Cell-free assay a. 12.3 *μ*M	ND	ND	[44]
				b. 19 *μ*M			
	b. Cordatolide B			In HOG.R5 cells a. 19.3 *μ*M	ND	a. 92 (200 *μ*g/mL)	[45]
				b. 11.7 *μ*M		b. 88 (200 *μ*g/mL)	
	*Calophyllum brasiliense/* GUT-70	Stem bark	Replication (quantification of p24 antigen)	8.44 *μ*M/3.48 *μ*M^*∗*^ (U1 cells (PMA))	ND	2.43 (U1 cells (PMA))	[46]
				8.44 *μ*M/4.32 *μ*M^*∗*^ (U1 cells (TNF-*α*))		1.95 (U1 cells (TNF-*α*))	
				>10 *μ*M/3.41 *μ*M^*∗*^ (Molt-4 cells)		>2.93 (Molt-4 cells)	
	*Ferula sumbul/* Imperatorin	Root	Replication	>100 mg/mL/<0.10 mg/mL^*∗*^	ND	>1000	[47]
	*Marila pluricostata/* a. Mesuol	Leaves and stem	Replication (luciferase activity)	a. 2 at 2.5 *μ*M	ND	ND	[48]
	b. Isomesuol			b. 2 at 2.5 *μ*M			
**Simple and related phenolic compounds**	*Humulus lupulus/* Xanthohumol	Hop	Cytopathic effect	0.82 *μ*g/mL (2 *μ*M)^*∗*^	8.82 *μ*g/mL (21.51 *μ*M)	10.8	[49]
			Replication (quantification of p24 antigen)	1.28 *μ*g/mL (3.21 *μ*M)^*∗*^			
			RT	0.50 *μ*g/mL (1.22 *μ*M)^*∗*^			
	*Thalassia testudinum/* Thalassiolin A	Whole plant	Integrase	2.1 *μ*M	ND	ND	[50]
			Strand transfer	0.4 *μ*M			
			Mg^+2^-dependent strand transfer	2 *μ*M			
			Replication (MAGI assay)	30 *μ*M			
	*Detarium microcarpum/* a. (−)-Epicatechin	Leaves	gp120	a. 2.0 *μ*g/mL	a. >100 *μ*g/mL	b. >100	[51]
	b. (−)-Epicatechin 3-O-gallate			b. 1.0 *μ*g/mL	b. >100 *μ*g/mL		
	*Camellia sinensis*	Commercial extract	RT	1.6 *μ*M	150 *μ*M	ND	[52]
	Epigallocatechin (EGCG)						
	*Scutellariae radix/* Baicalin monohydrate	Root	Replication (quantification of p24 antigen)	0.5 *μ*g/mL (PBMC infected with HIV-1)	ND	72% at 1 *μ*g/mL (PBMC infected with HIV-1) ▪	[53]
				0.2 *μ*g/mL (PBMC from HIV-1-infected individuals)		90% at 2 *μ*g/mL (PBMC from HIV-1-infected individuals) ▪	
			RT	2 *μ*g/mL	ND	50% at 2 *μ*g/mL ▪	
	*S*. *baicalensis* Georgi*/* Baicalin		Envelope	4 *μ*M	ND	ND	[54]
	Myricetin	Commercial extract	Replication (quantification of p24 antigen)	- 20.43 *μ*M (HIV-1 BaL in TZM-bl)	- 1214.72 *μ*M (TZM-bl)	ND	[55]
				- 22.91 *μ*M (HIV-1 MN in H9)	- 804.94 *μ*M (HeLa)		
				- 1.76 *μ*M (HIV-1 89.6 in H9)	- 1159.26 *μ*M (PBMC)		
				- 4.49 *μ*M (HIV-1 MN in PBMC)	- 1059.09 *μ*M (H9)		
				- 3.23 *μ*M (HIV-1 89.6 in PBMC)			
			RT	203.65 *μ*M			
	*Dioscorea bulbifera/* Myricetin	Bulbils	Integrase	3.15 *μ*M	ND	ND	[56]
	*Marcetia taxifolia/* Myricetin	Aerial parts	Replication (quantification of p24 antigen)	230 *μ*M^*∗*^	ND	ND	[57]
			RT	7.6 *μ*M	ND	ND	
	*Curcuma longa/* Curcumin	Roots	Processing 3′	95 *μ*M	ND	ND	[58]
			Strand transfer	40 *μ*M	ND	ND	
	a. *L-chicoric acid*	Synthetic	Processing 3′	a. 150 *μ*M	ND	ND	[59]
				b. 6 ± 1.5 *μ*M			
	b. Dicaffeoylmethane			c. 9 ± 7 *μ*M			
			Strand transfer	a. 140 *μ*M	ND	ND	
	c. Rosmarinic acid			b. 3.1 ± 0.1 *μ*M			
				c. 4 ± 1.5 *μ*M			
	*Curcuma longa/* a. Curcumin	Root	Replication (luciferase activity)	a. 0.7 *μ*M (CEM-T cells)/12 *μ*M (PBMC)	ND	ND	[60]
	b. Curcumin A			b. 0.8 *μ*M (CEM-T cells) and 2 *μ*M (PBMC)			
	*Curcuma longa/* L-chicoric acid	Synthetic	Integrase	500 nM at 10 *μ*M	ND	ND	[61]
			Entry	1 *μ*M			
**Proteins**	*Hericium coralloides/* *H*. *coralloides* laccase	Dried fruiting body	RT	0.06 *μ*M	ND	ND	[62]
	*Hericium erinaceum/* *H*. *erinaceum* laccase	Fresh fruiting body		9.5 *μ*M	ND	ND	[63]
	*Tricholoma mongolicum/* *T*. *mongolicum* laccase	Mycelium		0.65 *μ*M	ND	ND	[64]
	*Ganoderma lucidum/* *G*. *lucidum* laccase	Fresh fruiting body		1.2 *μ*M	ND	ND	[65]
	*Agaricus placomyces/* *A*. *placomyces* laccase	Fruiting body		1.25 *μ*M	ND	ND	[66]
	*Agrocybe cylindracea/* *A*. *cylindracea* laccase	Fresh fruiting body		12.7 *μ*M	ND	ND	[67]
	*Tricholoma giganteum/* *T. giganteum* laccase			2.2 *μ*M	ND	ND	[68]
	*Coprinus comatus/* *C*. *comatus* laccase	Mycelium		5.85 *μ*M	ND	ND	[69]
	*Pleurotus cornucopiae/* *P*. *cornucopiae* laccase	Fresh fruiting body		3.7 mM	ND	ND	[70]
				22 *μ*M	ND	ND	[71]
	*Clitocybe maxima/* *C*. *maxima* laccase			14.4 *μ*M	ND	ND	[72]
	*Lentinus tigrinus/* *L*. *tigrinus* laccase	Mycelium		2.4 *μ*M	ND	ND	[73]
	*Lentinus edodes/* *L*. *edodes* laccase	Fresh fruiting body		7.5 *μ*M	ND	ND	[74]
	*Agaricus bisporus/* *A. bisporus* lectin	Commercial lectin		1.9 *μ*M	ND	ND	[75]
	*Galanthus nivalis/* *G*. *nivalis* agglutinin	Bulbs	Cytopathic effect	0.4 *μ*g/mL^*∗*^	ND	ND	[76]
	*Hippeastrum hybrid/* *H*. *hybrid* agglutinin			0.4 *μ*g/mL^*∗*^	ND	ND	
	*Narcissus pseudonarcissus/* *N. pseudonarcissus* agglutinin			0.6 *μ*g/mL^*∗*^	ND	ND	
	*Listera ovata/* *L*. *ovata agglutinin*			0.3 *μ*g/mL^*∗*^	ND	ND	
	*Epipactis helleborine/* *E. helleborine* agglutinin	Leaves		0.04 *μ*g/mL^*∗*^	ND	ND	[77]
	*Cymbidium hybrid/* *C. hybrid* agglutinin			0.08 *μ*g/mL^*∗*^	ND	ND	
	*Myrianthus holstii/* *M. holstii* lectin	Root	Cytopathic effect	>50 *μ*g/mL/1.4 *μ*g/mL (150 nM) ^*∗*^	ND	ND	[78]
	*Oscillatoria agardhii/* *O. agardhii* agglutinin	Synthetic	Replication (quantification of p24 antigen)	24 ± 5 nM/0.51 *μ*M^*∗*^	>7 *μ*M (CEM-SS cells) and 0.51 *μ*M (MT-4 cells)	ND	[79]
			Cytopathic effect	13 ± 3 nM			
	*Griffithsia/* Griffithsin	Aqueous extract	gp120	0.04 nM^*∗*^ (HIV-1RF)-0.63 nM (HIV-1 T and M) ^*∗*^	ND	ND	[80]
	*Russula delica/* *R. delica* lectin	Fresh fruiting body	RT	0.26 *μ*M	ND	- 23.8% at 0.08 *μ*M ▪	[81]
						- 60.5% at 0.4 *μ*M ▪	
						- 94.2% at 2 *μ*M ▪	
	*Pleurotus citrinopileatus/* *P. citrinopileatus* lectin			0.93 *μ*M	ND	- 10% at 0.12 *μ*M ▪	[82]
						- 43.7% at 0.6 *μ*M ▪	
						- 90.3% at 3 *μ*M ▪	
	*Schizophyllum commune/* *S. commune* lectin			1.2 *μ*M	ND	ND	[83]
	*Pholiota adiposa/* *P. adiposa* lectin	Dried fruiting body		1.9 *μ*M	ND	ND	[84]
	*Inocybe umbrinella/* *I. umbrinella* lectin			4.7 ± 0.2 *μ*M	ND	ND	[85]
	*Boletus edulis/* *B. edulis* lectin	Fresh fruiting body		14.3 *μ*M	ND	ND	[86]
	*Hericium erinaceum/* *H. erinaceum* lectin	Dried fruiting body		31.7 *μ*M	ND	ND	[87]
	*Urtica dioica/* *U. dioica* agglutinin	Fruiting body	Cytopathic effect	0.9 *μ*g/mL^*∗*^	ND	ND	[77]
	*Flammulina velutipes/* a. Velutin	Fresh fruiting body	RT	a. ND	ND	ND	[88]
	b. Succinylated velutin			b. 5 *μ*g/mL			
	*Lyophyllum shimeji/* a. Lyophyllin	Fruiting body		7.9 nM	ND	ND	[89]
	*Hypsizygus marmoreus/* Hypsin			8 *μ*M	ND	ND	[90]
	*Hypsizygus marmoreus/* Marmorine			30 *μ*M	ND	ND	[91]
	*Luffa cylindrica/* Luffin P1	Seeds	Cytopathic effect	50 *μ*M^*∗*^	235 *μ*M	ND	[92]
			Replication (quantification of p24 antigen)	58 *μ*M^*∗*^			
	*Momordica balsamina/* Balsamin			10.2 nM	∼8.75 *μ*M	ND	[93]
	*Phytolacca americana/* a. PAP-I	Leaves		a. 14 nM	ND	ND	[94]
	b. PAP-II			b. 26 nM			
	c. PAP-III			c. 17 nM			
**Bromotyrosines**	Psammaplysine D	Marine sponge	Replication	ND	ND	51% at 0.1 *μ*g/mL ▪	[95]
	Aeroplysinin-1		Replication (reverse transcription assay)	3.76 *μ*M/2.5 *μ*M^*∗*^	ND	ND	[96]
			Replication (MAGI assay)	ND	ND	74% at 20 *μ*M ▪	[97]
			Late transcripts	ND	ND	48% at 10 *μ*M ▪	
			Nuclear import	ND	ND	67% at 10 *μ*M ▪	
	*Aiolochroia crassa/* Fistularine 3		Replication (reverse transcription assay)	10.3 *μ*M/6.9 *μ*M^*∗*^	ND	ND	[96]
	*Verongula rigida/* Purealidin B		Replication (MAGI assay)	ND	ND	57% at 80 *μ*M ▪	[97]
			Early transcripts	ND	ND	58% at 20 *μ*M ▪	
	*Psammaplysilla purpurea/* Mololipids mixture		Replication (SAR study)	>100 *μ*M/52.2 mM^*∗*^	ND	ND	[98]

**NC:** natural compound; **EC**_**50**_^*∗*^: effective concentration 50%; **IC**_**50**_**:** inhibitory concentration 50%; **CC**_**50**_: cytotoxic concentration 50%; **SI**: selectivity index; **RT**: reverse transcriptase; **ND**: nondetermined; ▪: percentage inhibition.

## Data Availability

No data were used in this study.
